# Points from Hospital Reports

**Published:** 1923-11

**Authors:** 


### POINTS FROM HOSPITAL REPORTS.

### Bradford Royal Infirmary.

A year's steady work unattended with any incident of a striking character is recorded in this report. A cursory inspection of the accounts discloses two remarkable things, an unusually large amount of annual subscriptions—£13,582 out of a total income of £35,000—and a total absence of legacies. Which of these phenomena is the more remarkable we will not undertake to decide; but much as we commiserate the Board of Management on the non-appearance of legacies in their account of income and expenditure, we congratulate them still more on the fine annual subscription list. It is a striking testimony to the financial skill of the administration, and a credit to the people of Bradford. We found the report of the Matron very interesting reading. While Miss Davies is evidently pleased to be able to say that in the senior examination every nurse passed, and in the final seven out of twelve obtained over 70 per cent. of the possible number of marks, she deplores the scarcity of suitable candidates for training, and concludes with these words: "It is becoming increasingly difficult to make a successful nurse of the present-day girl. The fact is, she lacks three of the essential qualities needed for nursing, the power of concentration, a sense of responsibility, and a keen interest in and love for work." Miss Davies is not the only Matron who has had to come to this conclusion.

### Miller General Hospital, Greenwich.

This is the report of a hospital thoroughly alive and cheerfully energetic in all its doings. We cannot recollect having read a more satisfactory and stimulating annual report. It begins by recording the election of an ideal President, in the person of Lord Queenborough, who combines in himself the qualities of chief advocate and most munificent benefactor—that invincible combination of precept and example—and concludes with thanks to every helper. If there is one thing more than another that has attracted our notice, it is the thorough-going way in which the people of the district which the hospital serves have bestirred themselves in its support. The patients themselves, the employees in the neighbourhood, the hospital's welfare society for Lewisham, Deptford and Greenwich, footballers, cricketers and school children—all seem to have vied with each other in efforts to aid the hospital. Not that they cannot do even better; we recognise that they can, and, such is the spirit that evidently animates them, we feel that they certainly will. Of the work of the year no more need be said than that, of the hospital's 100 beds, 93 were kept regularly occupied throughout the year, and that out-patients' attendances numbered 129,000, of which 75,000 were casualties—very large figures in view of the number of beds. Turning to the accounts, we rejoice to find that the income of the year sufficed, not only to meet the expenditure, but to wipe out the deficiency of 1921. Passing on to expenditure, we observe a reduction of more than £3,000, modestly attributed exclusively to the fall in the price of commodities.

### Nottingham General Hospital.

Whatever worries afflict the authorities of Nottingham's General Hospital, an uphill struggle to make ends meet is certainly not among them. Indeed, so far from that being the case, we find that the ordinary income for the year was sufficient, not merely to provide for the ordinary expenditure, but to leave a balance of some 11 per cent. This satisfactory state of affairs is mainly due to the very remarkable success of the Hospital Saturday Fund organisation in the town, and that too at a time when depression in the lace manufacture prevailed of such a serious character that the Secretary of the Lace Federation was recently reported in the *Times* to have said "that the whole industry stood on the brink of a great disaster." During last year many thousands in Nottingham must have been forced by the inexorable pressure of circumstances to curtail their expenditure, and, to their honour be it said, it was not their contributions to the hospital that they decided first to lop. The spirit of self-help is evidently very much alive in Nottingham, for not only does the hospital Saturday organisation contribute to the hospital £27,000, which is 50 per cent. of its ordinary income, but as many as 140 entertainments for the hospital were organised during the year, producing in round figures £2,700, while patients' payments furnished £1,500. Letters from patients, a selection from which is included in the report of the Monthly Board, show that the quality of the work of the hospital is such as fully to merit support such as we have described. The report itself is a production which it were churlish not to praise. Text, paper, type and illustrations are admirable—in short, a model of what such a publication should be.

